# Global patterns of plant and microbial biomass in response to CO_2_ fumigation

**DOI:** 10.3389/fmicb.2023.1175854

**Published:** 2023-04-20

**Authors:** Junliang Zou, Weiwei Zhang, Yun Zhang, Juying Wu

**Affiliations:** Institute of Grassland, Flowers, and Ecology, Beijing Academy of Agriculture and Forestry Sciences, Beijing, China

**Keywords:** rising CO_2_ level, plant biomass, soil microbial biomass, fungal biomass, bacterial biomass, meta-analysis

## Abstract

**Introduction:**

The stimulation of plant and microbial growth has been widely observed as a result of elevated CO_2_ concentrations (eCO_2_), however, this stimulation could be influenced by various factors and their relative importance remains unclear.

**Methods:**

A global meta-analysis was performed using 884 lines of observations collected from published papers, which analyzed the eCO_2_ impact on plant and microbial biomass.

**Results:**

A significant positive impact of eCO_2_ was observed on various biomass measures, including aboveground biomass (20.5%), belowground biomass (42.6%), soil microbial biomass (10.4%), fungal biomass (11.0%), and bacterial biomass (9.2%). It was found that eCO_2_ levels above 200 ppm had a greater impact on plant biomass compared to concentrations at or below 200 ppm. On the other hand, studies showed that positive effects on microbial biomass were more prominent at lower eCO_2_ levels (≤200 ppm) than at higher levels (>200 ppm), which could be explained by soil nitrogen limitations. Importantly, our results indicated that aboveground biomass was controlled more by climatic and experimental conditions, while soil properties strongly impacted the stimulation of belowground and microbial biomass.

**Discussion:**

Our results provided evidence of the eCO_2_ fertilization effect across various ecosystem types, experimental methods, and climates, and provided a quantitative estimate of plant and soil microbial biomass sensitivity to eCO_2_. The results obtained in this study suggest that ecosystem models should consider climatic and edaphic factors to more accurately predict the effects of global climate change and their impact on ecosystem functions.

## Introduction

Starting from the Industrial Revolution, human activities have been altering the composition of the atmosphere on a global scale. This has led to a significant disturbance in the functioning of terrestrial ecosystems, which provide crucial services that support human life and health, such as carbon storage to combat climate change and food security. The atmospheric CO_2_ concentration has risen by almost 50% compared to preindustrial levels (Legg, 2021). The growing concerns about the effects of elevated levels of atmospheric carbon dioxide (eCO_2_) have boosted research on ecosystem processes, including plant biomass production and global biogeochemical cycles (Reich et al., 2006b; Terrer et al., 2021). The effects of eCO_2_ on plant biomass production have been widely studied, but the response of different plant components, such as aboveground and belowground biomass, as well as associated microbial communities, is still a topic of debate.

In the last 30 years, many studies have aimed to measure how plants respond to increasing levels of atmospheric carbon dioxide by exposing them to eCO_2_. In the global carbon cycle, terrestrial ecosystems are essential because they sequester around one-third of the CO_2_ emissions induced by human activities (Legg, 2021). Elevated CO_2_ concentrations can lead to an increase in plant biomass production and subsequent carbon sequestration. This can occur through direct improvements in photosynthesis as well as indirect improvements in resource use efficiency, such as water and nitrogen (Luo et al., 2006; Zou et al., 2020; Xia et al., 2021). Studies have shown varying results on the effects of eCO_2_ on plant biomass production. Nutrient and water limitations, as well as acclimation, can contribute to inconsistent results. Elevated CO_2_ levels can exacerbate nutrient limitations and may not increase plant biomass production in water-limited ecosystems (Norby et al., 2005; Reich et al., 2006a). Additionally, plants may adjust their physiology and allocation patterns in response to elevated CO_2_ levels, leading to no significant increase in aboveground biomass production (Drake et al., 2011). Although a number of variables, including the landuse type, the experimental design, and the climate have been recognized as control factors that can regulate the response of plant biomass to eCO_2_ (Luo et al., 2006), it is still unknown how significant any of these variables is, which creates uncertainty in the projections for future climates.

Although much of the research on ecological reactions to global climate change has focused on plants (Peterson et al., 1999; Ainsworth and Rogers, 2007; Wang et al., 2012; Du et al., 2019; Cui et al., 2020; Wang et al., 2022), research studies examining the influences of global climate change on soil microorganisms has been increased in recent years (Sun et al., 2021; Li et al., 2022; Lin et al., 2022; Peng et al., 2022). Similar to plants, soil microorganisms are sensitive to global changes such as rising atmospheric CO_2_ concentrations. As a result, changes in the community structure and diversity of microorganisms can impact ecosystem processes that are influenced by these microorganisms (Garcia-Palacios et al., 2015). The complex, diversified communities that makeup soil biota can alter in abundance, composition, and physiology as a result of climate change (Eisenhauer et al., 2012; Li et al., 2022). Diverse results have been reported on the responses of soil microbial biomass (including fungal biomass and bacterial biomass) to eCO_2_ (Gorissen et al., 1995; Hu et al., 2001; Yang et al., 2021), which could be attributed to differences in experimental design, including varying levels and durations of eCO_2_ exposure (Reich et al., 2006b), as well as different climatic conditions (Yue et al., 2017) and landuse types (Luo et al., 2006). The methods employed for CO_2_ enrichment, which might produce distinct microclimates in the soil, further complicate how eCO_2_ affects soil microbial biomass (Huang et al., 2017). Different CO_2_ concentrations used in CO_2_ fumigation experiments play a critical role in regulating soil microbial biomass (Yang et al., 2021; Li et al., 2022), as high CO_2_ levels can promote plant nutrient uptake, leading to reduced soil nutrient availability and lower microbial abundance (Luo et al., 2004; Xiao et al., 2017; Zou et al., 2020; Terrer et al., 2021). Abundance measurements are commonly used to assess soil biota responses to eCO_2_ and are easier to standardize across studies and taxa (Treseder, 2004, 2008; Blankinship et al., 2011). In controlled environments, where nutrient limitations are absent, the response of soil microbial biomass to eCO_2_ may be positive, unlike in natural ecosystems (Hu et al., 2017). Despite extensive research on the effects of eCO_2_ on soil microbial biomass, our understanding of the underlying factors and their relative importance is still limited.

The effects of eCO_2_ on plant biomass production and associated microbial communities are complex and vary depending on several factors. Therefore, further studies are needed to better understand the mechanisms behind these effects and to refine hypotheses regarding the response of different plant components and associated microbial communities to eCO_2_. In this study, we conducted a meta-analysis of data from various global change experiments to assess how significant the effects of eCO_2_ are on plant and microbial biomass and identify the factors that regulate these effects. Our specific objectives were to: (i) quantify the magnitude of the effects of elevated CO_2_ on above-and belowground plant biomass, as well as total microbial, fungal, and bacterial biomass; and (ii) assess the relative significance of the factors that regulate these responses to elevated CO_2_.

## Materials and methods

### Data collection

**Figure 1:**
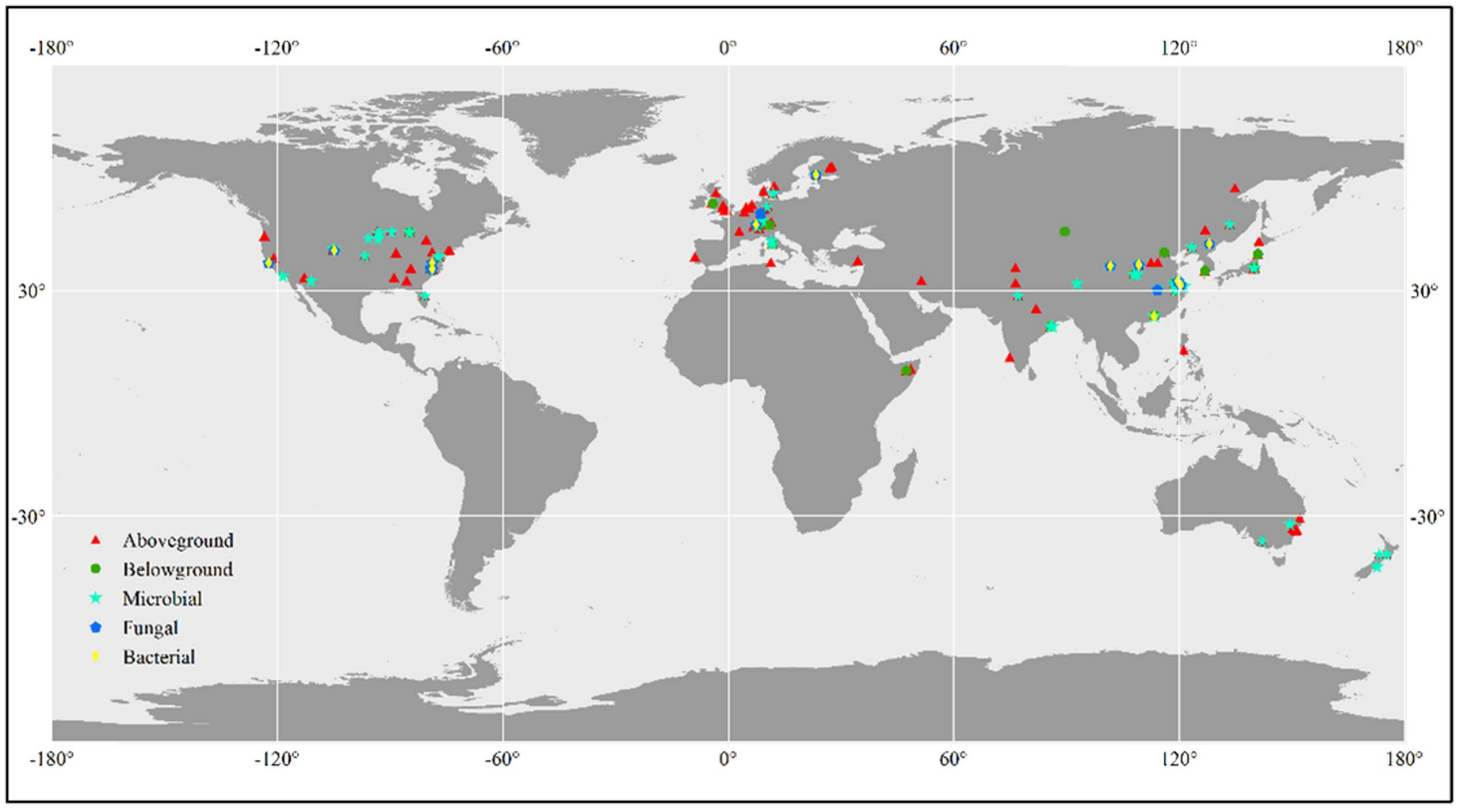
Global distribution of the study sites.

We conducted a literature search using the Web of Science,^1^ Google Scholar,^2^ and CNKI (China National Knowledge Infrastructure)^3^ to identify relevant studies. The search terms we used were “CO_2_ fumigation” or “elevated CO_2_” or “CO_2_ enrichment” or “rising CO_2_ concentration” or “carbon dioxide” and “total biomass” or “plant production” or “aboveground biomass” or “belowground biomass” or “plant biomass” or “microbial biomass” or “fungal biomass” or “bacterial biomass” or “microbial abundance” or “microbial community.” Studies had to adhere to the following standards in order to be considered for our analysis: (1) experimental design including the experimental method has been reported; (2) last at least one growing season; (3) initial species composition between control and treated plots should be no difference; and (4) means, standard deviation, and sample sizes have been reported or can be calculated. For multifactor global change experiments, we only considered the control and CO_2_-fumigated plots that were subject to equivalent experimental conditions. For individual experiments that obtained multiple measurements on the same variable, we included only the most recent data. Web Plot Digitizer^4^ was used to obtain data from graphs. In total, 844 lines of observations reporting plant and microbial biomass results from global terrestrial ecosystems, mainly aboveground biomass (443 lines of observations), belowground biomass (98 lines of observations), microbial biomass (207 lines of observations), fungal biomass (47 lines of observations), and bacterial biomass (49 lines of observations), were included in the meta-analysis (Figure 1). The observations were categorized according to the following three factors: CO_2_ magnitude (≤200, and *>*200 ppm), landuse type (cropland, forest, and grassland), experimental method [(Free-Air CO_2_ Enrichment) FACE, (Open-Top Chamber) OTC, and (Growth Chamber) GC].

We collected various environmental and experimental factors for each experiment in our dataset, including latitude, longitude, mean annual temperature (MAT), mean annual precipitation (MAP), CO_2_ concentration change (ΔCO_2_), land use type, pH, soil organic carbon content (SOC), soil total nitrogen content (TN), and carbon: nitrogen ratio (CN). If not reported in the reference, we obtained data on MAT and MAP from the WorldClim database.^5^ To calculate the aridity index (AI), we divided the annual precipitation by potential evaporation, which we obtained from the same database. For SOC and TN (0–20 cm depth), we used the HSWD 2.0 database^6^ if data were not reported in the reference. In total, we included 12 factors in our research as predictors of eCO_2_ impacts (see Supplementary Table S1).

### Meta-analysis

For each individual observation, the calculation of the response ratio (RR) and its variance (V_RR_) was performed using the natural logarithm transformation. Further details on these calculations can be found in (Hedges et al., 1999; Zou et al., 2020).


(1) $$RR=\ln\left(\frac{{\bar{X}}_{e}}{{\bar{X}}_{c}}\right)$$

(2) $$V_{RR}=\frac{s_{e}^{2}}{n_{e}\times {\left({\bar{X}}_{e}\right)}^{2}}+\frac{s_{c}^{2}}{n_{c}\times {\left({\bar{X}}_{c}\right)}^{2}}$$

The variables ${\bar{X}}_{e}$ and ${\bar{X}}_{c}$ represent the mean values of each variable for the plots exposed to eCO_2_ and ambient conditions, respectively. The corresponding number of replicates for the eCO_2_ and ambient treatment are *n_e_* and *n_c_*, respectively. The sample standard deviations for eCO_2_ and ambient treatment are represented by *s_e_* and *s_c_*, respectively.

Weighting factor (*w_ij_*), weighted mean response ratio (*RR*_++_), and confidence interval (95% CI) were calculated as follows:


(3) $$w_{ij}=\frac{1}{V_{RR}}$$

(4) $$RR_{++}=\frac{\sum_{i=1}^{m}\sum_{j=1}^{k}w_{ij}RR_{ij}}{\sum_{i=1}^{m}\sum_{j=1}^{k}w_{ij}}$$

(5) $$s\left(RR_{++}\right)=\sqrt{\frac{1}{\sum_{i=1}^{m}\sum_{j=1}^{k}w_{ij}}}$$

(6) $$95\%\mathrm{CI}=RR_{++}\pm 1.96s\left(RR_{++}\right)$$

The within-study variance and the between-study variance, which are caused by sampling errors and changes in experimental circumstances, are the two variables that make up the weighted mean effect size in the random effects model. Observations with smaller variances are given more weights (Borenstein et al., 2009). This was carried out using MetaWin 2.1 (Rosenberg et al., 2000) for meta-analysis, while the moderators employed in fitting random effects models were land use type, method, and CO_2_ magnitude. Significant effects of eCO_2_ concentrations on plant and microbial biomass variables were determined if the 95% confidence interval for the response ratio did not overlap with 0. Percentage changes were estimated using the formula (exp^RR++^ − 1) × 100%. We conducted random forest analysis to quantify the relative importance of 12 predictors to biomass by using the “randomForest” package in R (Chen et al., 2019).

## Results

**Figure 2:**
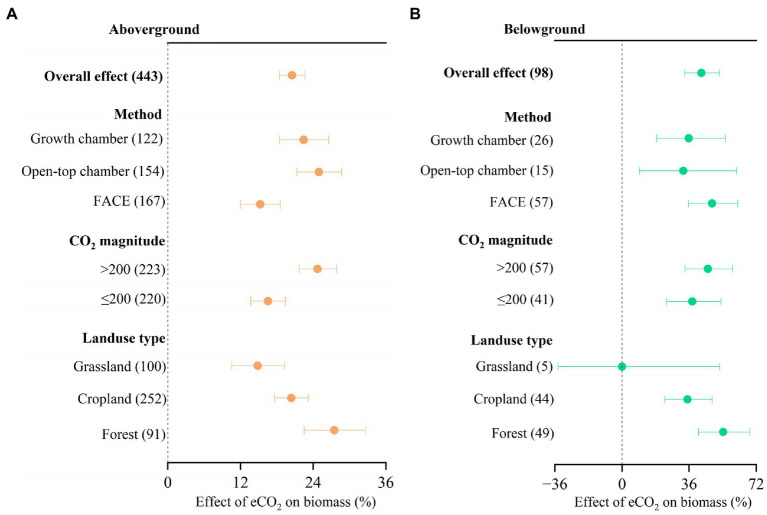
Meta-analysis of the effect of eCO_2_ on aboveground biomass **(A)** and belowground biomass **(B)** across different factors. Error bars represent 95% confidence intervals; sample sizes are shown in parentheses. FACE, Free-Air CO_2_ Enrichment.

We found that eCO_2_ significantly increased aboveground biomass by 20.5% (Figure 2, 95% confidence interval: 18.4–22.6%) and belowground biomass by 42.6% (33.6–52.2%). There was no significant difference in CO_2_ response between different experimental methods, CO_2_ magnitude, and landuse type, respectively, for belowground biomass (Figure 2B). However, there were significant differences in CO_2_ response between OTC (24.9, 95% confidence interval: 21.3–28.7%) and FACE (15.3, 95% confidence interval: 12.0–18.6%) methods for aboveground biomass (Figure 2A). Significant differences in CO_2_ response between forest (27.5, 95% confidence interval: 22.5–32.7%) and grassland (14.8, 95% confidence interval: 10.6–19.3%) were also observed for aboveground biomass. The increase in aboveground biomass was larger at high ΔCO_2_ concentration (*>*200 ppm) than at low ΔCO_2_ concentration (≤200 ppm; Figure 2A).

**Figure 3:**
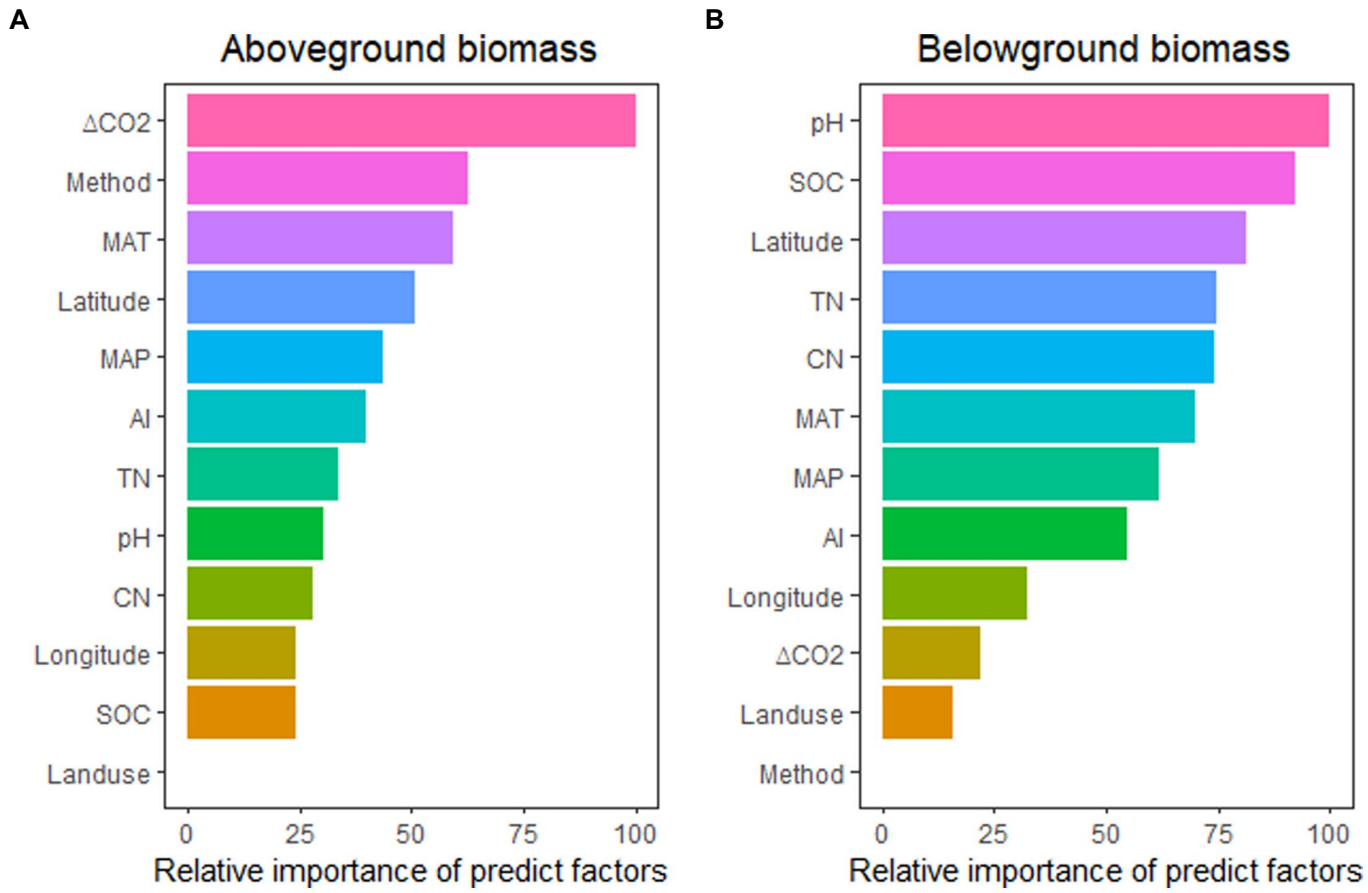
The relative importance of predictors for the effect of eCO_2_ on aboveground biomass **(A)** and belowground biomass **(B)**. MAT, mean annual temperature; MAP, mean annual precipitation; ΔCO_2_, CO_2_ concentration change; SOC, soil organic carbon content; TN, soil total nitrogen content; CN, SOC: TN ratio; AI, aridity index.

The impacts of eCO_2_ on above-and belowground biomass were different for each of these factors (Figure 3), with aboveground biomass predicted better by ΔCO_2_ concentration, experimental method, MAT, Latitude, and MAP, while belowground biomass predicted better by pH, SOC, Latitude, TN, and CN ratio. These relationships indicated that the stimulation of aboveground biomass was controlled more by climatic and experimental conditions, while soil properties controlled belowground biomass stimulation.

**Figure 4:**
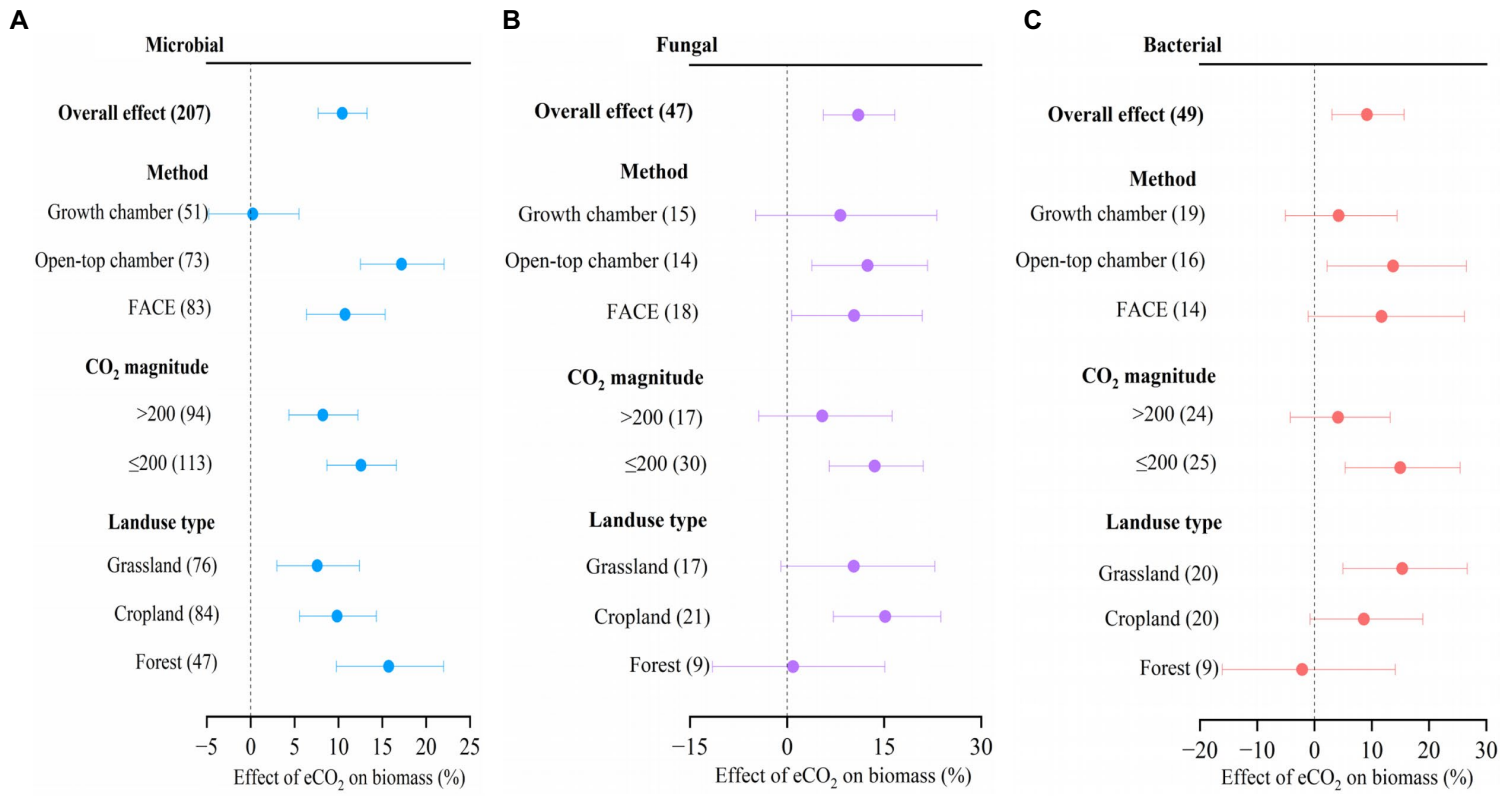
Meta-analysis of the effect of eCO_2_ on microbial biomass **(A)**, fungal biomass **(B)**, and bacterial biomass **(C)** across different factors. Error bars represent 95% confidence intervals; sample sizes are shown in parentheses. FACE, Free-Air CO_2_ Enrichment.

We found that eCO_2_ significantly increased soil microbial biomass by 10.4% (Figure 4, 95% confidence interval: 7.7–13.3%), fungal biomass by 11.0% (5.6–16.7%), and bacterial biomass by 9.2% (3.0–15.7%), respectively. Across all the studies, there was no significant difference in CO_2_ response between different experimental methods, between the two CO_2_ magnitudes, and between different landuse types, respectively, for microbial, fungal, and bacterial biomass (Figure 4). When ΔCO_2_ concentration was ≤200 ppm, the microbial, fungal, and bacterial biomass were significantly increased by 12.6% (8.7–16.6%), 13.5% (6.5–21.0%), and 15.0% (5.4–25.4%), respectively, while only significant increase was observed for microbial biomass (8.2%) at high ΔCO_2_ concentration (*>*200 ppm; Figure 4).

When compared to the OTC method, the application of the FACE method resulted in a notable rise in microbial biomass (17.2%), as well as in fungal biomass (12.4%) and bacterial biomass (13.7%). There was no significant effect of eCO_2_ on microbial biomass, fungal biomass, and bacterial biomass by the method of growth chamber. Moreover, significant increases in microbial biomass (10.7%) and fungal biomass (10.4%) were observed by the method of FACE (Figure 4).

Microbial biomass was increased by 7.6% (3.0–12.4%) in grassland, 9.9% (5.6–14.3%) in cropland, and 15.7% (9.8–22.0%) in forest, respectively in response to eCO_2_. Fungal biomass was increased by 15.2% (7.2–23.8%) in cropland, and bacterial biomass increased by 15.3% (5.0–26.7%) in grassland, respectively in response to eCO_2_ (Figure 4).

**Figure 5:**
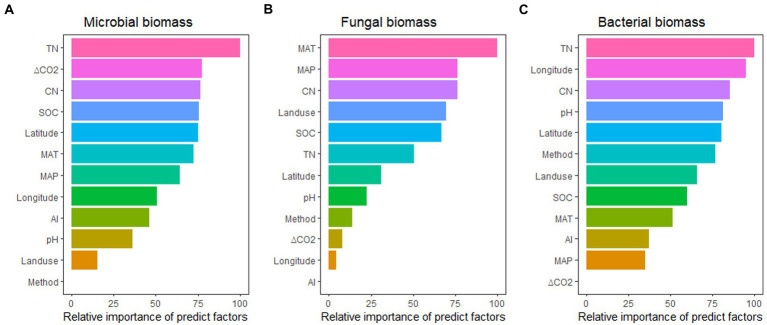
The relative importance of predictors for the effect of eCO_2_ on microbial biomass **(A)**, fungal biomass **(B)**, and bacterial biomass **(C)**. See Figure 3 for abbreviations.

Across these variables, the effects of eCO_2_ on soil microbial biomass were better predicted by TN, ΔCO_2_, CN ratio, SOC, and latitude; the effects of eCO_2_ on fungal biomass were better predicted by MAT, MAP, CN ratio, landuse type, and SOC; while the effects of eCO_2_ on bacterial biomass were better predicted by TN, longitude, CN ratio, pH, and latitude. It seems that N availability (TN/CN ratio) seems to have a dominant role in regulating the impact of eCO_2_ on total microbial, fungal, and bacterial biomass (Figure 5).

## Discussion

In total, 844 lines of observations presenting findings around the globe were included in the database we used for our research (Figure 1; Supplementary Table S1). The majority of the experiments were carried out in the northern hemisphere. The ranges of CO_2_ concentration changes are consistent with the projections of the end of the century, which were primarily between 100 and 500 ppm (Supplementary Table S1). Through our empirical evidence-based meta-analysis, we present a global-scale evaluation of the impact of eCO_2_ on plant and soil microbial biomass. Our findings are generally consistent with prior studies that have reported increased biomass production and microbial biomass in response to eCO_2_ (De Graaff et al., 2006; Luo et al., 2006; Wang et al., 2012; Garcia-Palacios et al., 2015; Terrer et al., 2021). Our analysis revealed that plant aboveground biomass production is primarily driven by climatic and experimental conditions, whereas belowground /microbial biomass is more influenced by soil properties such as TN/CN, pH, and SOC (Figure 3; Figure 5).

Rising atmospheric CO_2_ concentration generally stimulates photosynthesis by 30–70% (Wang et al., 2012), and the consequent outcome of increased plant biomass production is estimated to be around 30% according to Luo et al. (2006) and Terrer et al. (2021). This study reported a 20.5% increase in aboveground biomass under elevated CO_2_, which is similar to these estimates. Notably, our meta-analysis revealed that eCO_2_ increased aboveground biomass by 24.9% in OTC experiments, which is significantly higher than the increase in FACE experiments (15.3%). Higher ΔCO_2_ concentration (*>*200 ppm) showed to have a larger impact on plant biomass than at lower ΔCO_2_ concentration (≤200 ppm). Additionally, aboveground biomass was substantially more affected by eCO_2_ in forests than in grasslands (Figure 2A). The mechanisms underlying the observed differences in the effect of eCO_2_ on aboveground biomass between OTC and FACE experiments, as well as between different ecosystems, are complex and not yet fully understood. However, several hypotheses have been proposed based on experimental evidence. One possible explanation for the greater effect of eCO_2_ on aboveground biomass in OTC experiments compared to FACE experiments is that OTC experiments provide a more controlled environment with less variability in CO_2_ concentrations and other environmental factors. This may lead to a more consistent and larger response of plants to eCO_2_, as they are not subject to the same level of environmental fluctuations and stressors as in FACE experiments (Norby and Zak, 2011). Additionally, OTC may alter microclimatic conditions such as temperature and humidity, which may further amplify the response of plants to eCO_2_ (Norby et al., 2001). The possible mechanism for the greater effect of eCO_2_ on aboveground biomass at higher ΔCO_2_ concentrations (>200 ppm) is that plants may reach a saturation point in their response to CO_2_ enrichment at lower levels. This saturation point may vary among plant species and ecosystems, and may be influenced by factors such as nutrient availability and water availability (Norby et al., 2005). Finally, the greater effect of eCO_2_ on aboveground biomass in forests compared to grasslands may be because that compared to grasses, forest trees have lower nutrient uptake efficiency and stronger competition with soil microbes (Kaye and Hart, 1997; Cheng and Bledsoe, 2004). Whereas other research reported that the sensitive response of forest fine roots to high CO_2_ may increase nutrient uptake efficiency and heighten competition with soil microbes (Nambiar and Sands, 1993; Dybzinski et al., 2011).

Previous research reported that eCO_2_ stimulated the root biomass production by ~28.3% (De Graaff et al., 2006), which is lower than the increase reported in our study. Roots are crucial in controlling ecosystem carbon (C) and nitrogen (N) cycling, and rhizodeposition could increase soil C due to increased root biomass (Bader et al., 2009). Because root-derived materials offer an immediate substrate for microbial activity, increased CO_2_ probably has a more direct impact on soil C and N cycling through root-derived materials than aboveground litter decomposition (Zak et al., 2000). The allocation of belowground biomass by plants and alterations in C distribution in ecosystems are crucial in predicting future plant responses to rising atmospheric CO_2_ (Cotrufo and Gorissen, 1997; Iversen, 2010). According to our results, the stimulation of eCO_2_ on root biomass is stronger than aboveground biomass (Figure 2), which is thought to be due to increased photosynthesis and carbon allocation to belowground tissues as well as changes in nutrient availability and uptake (Norby and Zak, 2011). In order to meet the growth demand of nutrients under eCO_2_, plants tend to increase the growth of deeper roots which were favor the ability to absorb nutrients and sequester more carbon (Cotrufo and Gorissen, 1997; De Graaff et al., 2006; Iversen, 2010). Previous research reported inconsistent results that root may not always present higher biomass production compared to shoot biomass production (Nowak et al., 2004). Since data regarding simultaneous measurements of root and shoot biomass is still extremely limited, it is still difficult to definitively answer the question regarding the relative response of roots and shoots to eCO_2_.

Our results showed that eCO_2_ had a favorable influence on bacterial and fungal biomass (Figures 4B,C), which is similar to the stimulation of total soil microbial biomass that have been widely reported in literature (Eisenhauer et al., 2012). This is likely due to the increased input of soil carbon resulting from above-and belowground litterfall under the conditions of elevated CO_2_ concentrations. However, our synthesis reveals that this trend only holds true when the increase in CO_2_ concentration (ΔCO_2_) is below 200 ppm, indicating that no significant impacts were observed beyond this threshold level (Figure 4). N limitation induced by high eCO_2_ treatment levels is a probable explanation for this observation (Oren et al., 2001; Luo et al., 2004; Norby et al., 2010; Zou et al., 2020).

Initially, improved carbon (C) availability likely benefits microbes, but their biomass turnover is relatively rapid (Zak et al., 2000; Heath et al., 2005; Lukac et al., 2009). Additionally, increased N immobilization in growing plant biomass (Luo et al., 2004) may also limit microbial growth (Hu et al., 2001, 2006). Our results highlight the crucial role of N availability in controlling soil microbial biomass (Figure 5). Moreover, eCO_2_ generally increases the CN ratios as the carbon pool increased larger than N pools (Luo et al., 2006; Zou et al., 2020; Xia et al., 2021), which reduces the N availability and leads to the acclimation of microbial biomass growth to high levels of CO_2_. Therefore, microbial biomass did not show significant increase under high ΔCO_2_ concentration (>200 ppm) despite the increase in plant biomass production and associated C inputs. In addition, previous research by Dieleman et al. (2010) showed that eCO_2_ reduced the mineral N or N mineralization, but contradict results has also been reported that mineral N or N mineralization being promoted under eCO_2_ conditions. Therefore, more work is needed to investigate the N availability and transformation in order to fully understand how eCO_2_ influence the N processes and further the plant growth. Our findings suggest that eCO_2_ makes plants more effective at immobilizing N, resulting in microbial growth becoming N-limited as usually larger C inputs typically result in more N uptake, even in ecosystem where the N is insufficient (Finzi et al., 2007). The stimulation of soil microbial biomass by eCO_2_ observed in our study in OTC experiments (Figure 3A), which regulates microclimate (Huang et al., 2017). A previously published meta-analysis has described the different impact of CO_2_ fertilization on plant and soil microbial biomass in various ecosystem types, including croplands, forests, and grasslands (Li et al., 2022). However, we found that plant and soil microbial biomass in certain ecosystems showed to have no significant responses to eCO_2_ as insufficient data were available for these ecosystems (Figures 2A, 4B,C).

## Conclusion

Our study indicates that increasing atmospheric CO_2_ concentrations will significantly affect both plant growth and soil microbes. The effects of eCO_2_ on plant and microbial biomass were found to be dependent on the specific eCO_2_ level. The stimulation of aboveground biomass was more influenced by climatic and experimental factors, while the stimulation of belowground biomass was more influenced by soil characteristics. Our findings underscore the crucial role of N availability in regulating microbial responses to elevated CO_2_ concentration. Our synthesis provided empirical evidence on the impact of CO_2_ fertilization across a range of ecosystem types, experimental methods, and climates. Furthermore, it provided a quantitative estimate of plant and soil microbial biomass sensitivity to eCO_2_, which can aid in predicting soil microbial responses to future increases in atmospheric CO_2_ concentrations.

## Data availability statement

The original contributions presented in the study are included in the article/Supplementary material, further inquiries can be directed to the corresponding author.

## Author contributions

JZ, WZ, YZ, and JW designed the experiments. JZ and JW collected and analyzed data. All authors wrote the article, contributed critically to the draft, and approved the final version of the manuscript for publication.

## Funding

This work was financially supported by the Excellent Youth Scholars Program and the Special Project on Hi-Tech Innovation Capacity (KJCX20210416) from the Beijing Academy of Agriculture and Forestry Sciences (BAAFS).

## Conflict of interest

The authors declare that the research was conducted in the absence of any commercial or financial relationships that could be construed as a potential conflict of interest.

## Publisher’s note

All claims expressed in this article are solely those of the authors and do not necessarily represent those of their affiliated organizations, or those of the publisher, the editors and the reviewers. Any product that may be evaluated in this article, or claim that may be made by its manufacturer, is not guaranteed or endorsed by the publisher.

## Footnotes

^1^ https://apps.webofknowledge.com/

^2^ https://scholar.google.com/

^3^ https://www.cnki.net/

^4^ https://apps.automeris.io/wpd/

^5^ http://www.worldclim.org/

^6^ https://gaez.fao.org/pages/hwsd
